# The Missing or Suppressed Teeth in Man. Inaugural Address

**Published:** 1891-12

**Authors:** Andrew Wilson


ARTICLE III.

### THE MISSING OR SUPPRESSED TEETH IN MAN. INAUGURAL ADDRESS.

BY ANDREW WILSON, ESQ., L. D. S.

Delivered before the Edinburgh Dental Students' Society, 2nd November, 1891.

At our social gathering of last session, some of you will probably remember that in the few remarks I made, I strongly urged the advisability, or rather the necessity, of acquiring habits of accurate observation, mentioning in general terms, as an example of the result of not doiug so, the case of a paper on a subject in comparative dental anatomy, "Teeth in Rabbits," by an eminent American dentist, which had a very liberal supply of errors of that description. Even in one of the illustrations, the lower jaw was reversed, and premolars were figured and described as molars, molars as premolars, and altogether the figure, a vertical section of part of the lower jaw and cheek-teeth, shewed what by no possibility could be seen, while the very characteristic arrangements of the dental tissues in these teeth were altogether overlooked. Some of the conclusions come to are correspondingly sweeping, thus he discovered that, all previous observers to the contrary notwithstanding, "these teeth are coated with enamel only upon their anterior surfaces," and that "the structure of the enamel differs from that of the incisors, in as much as it is composed of two layers." An inspection of the beautiful and accurate figure of a transverse section given in "Owen's Odontography," would, I suspect, have led to the suppression of the paper.

Now a great deal of literary dental work, consisted of recording of observed facts, from which we are all too apt to construct hypothesis, it followed that any errors of observation made what they believed facts, fictions, and so, as in the paper referred to, any generalizations or conclusions based on them were vitiated. As shewing how much observations, entered into scientific dental questions, I will, with your permission, take a subject with which I have been a good deal mixed up, that of the missing or suppressed teeth in man. What was known as the typical dentitions in placental mammals was I.3-3, C.1-1, PM.4-4, M.3-3, while that in man was I.2-2, C.1-1, PM.2-2, M.3-3, so that in man, one incisior and two premolars were missing, the question to be determined being, which of the incisiors and premolars these were.

Up till comparatively lately, the unquestioned decision was, to quote from Mr. Tomes' Manual, 2nd edition, 1882, "The human subject does not possess the third incisor, nor the first two premolars," then he goes on to add the deduction "so that a somewhat abrupt change of form, in passing from the incisors to the canines, and from the latter to the bicuspids is no more than might be anticipated," no notice being taken of the at least as abrupt step from the premolars to the molars, although according to his view the second bicuspid was the fourth premolar, so no tooth was missing there.

This view so decidedly expressed, I accepted, until when on the formation of the Edinburgh Dental School, I became lecturer on Dental Anatomy, and so was necessitated to go more thoroughly into the subject.

This led first to my differing as to the missing incisor, and then as my own observations extended, as to that of the premolars also; and for several years I have been teaching that the missing incisor is the middle or second, and the premolars are the first and fourth.

My reasons for so concluding were purely dental, and may be condensed into the following three. First, teeth which are liable to be suppressed, not unfrequently shew themselves either as dwarfed, or as more or less rudimentary in form, that is conoid; second, the corollary of this, teeth which are normally suppressed in any species, not unfrequently put in an appearance, usually in a more or less rudimentary form; lastly, I recognized those teeth which in man are called supernumerary or supplemental, as being the normally missing members of the typical series.

Now, it is very difficult to upset old-established conclusions, more especially when these are supported by great names, but, fortunately the question as to the missing incisor has got mixed up with that as to the situation of the fissure on the alveolar border, in cases of maxillary cleft.

Most of you are aware that such fissure (not necessarily involving cleft palate) was held to be due to arrest of development of the inter-maxillary bone, and that the fissure on the alveolar process was in the line of maxillo-intermaxillary suture.

But closer observations showed that, in very many cases, there was present on the distal side of the fissure, and so on the mesial side of the canine, a more or less abnormally formed tooth, which undoubtedly belonged to the incisor series.

This necessarily involved that one or other of two received views was wrong, either the definition of the canine as being the tooth at the mesial extremity of the maxillary bone, that is, close to or at the suture, or that as to the position of the fissure as being uniform. Dr. Paul Albrecht, an eminent foreign anatomist, as the result of an extensive series of observations, both human and comparative, adopted the latter view, and came to the conclusion that the inter-maxillary bone originally consisted of two bones; the mesial one of which, in man normally carried the socket of the central incisor, the distal, that of the lateral, and that the fissure not unfrequently lay in their line of junction, that was, it was intra-incisive, also, that in those cases of cleft in which an extra incisor was present, its socket was invariably in the mesial portion, along with that of the central, showing it to be the second incisor of the typical dentition.

Professor Sir William Turner, in a paper on "The relation of the alveolar form of cleft palate to the incisor teeth and the intermaxillary bones," read before the Royal Society of Edinburgh, strongly supported Dr. Albrecht's conclusions, and proposed that the tooth between the fissure and the canine should previously receive the name of pre-canine, although, in his own opinion, it was undoubtedly the third incisor of the typical dentition and the normal lateral in man.

Mr. Oakley Coles, who had had very considerable experience in cleft palate cases, exhibited a large series of models of such before the Odonto-Chirurgical Society, and while pointedly declining to commit himself as to which incisor was that suppressed, stated that "It will be observed that there is not a single instance of fissure of the alveoli occurring between a true lateral incisor and canine," in the cases he had met with.

As a consequence of this discussion, Mr. Tomes, in the latest edition of his Manual, closing a paragraph referring to the facts I have been laying before you, says, "if this inference be correct, then the lost incisor in man is probably I2," and treating of the teeth of the primates, says, we "bring forward a certain amount of evidence which raises a doubt as to whether it is not I2 which is missing in man."

As regards the missing premolars, although we have not got to go so far back among mammals to find a nearer approach to the typical number, than seen in man, all the American monkeys have PM3-3; there is less advance.

All my observations, both human and comparative, lead me to the conclusion that those suppressed are the typical PM.1 and PM.4. No one claims PM.1 as present in man, so that the question is limited to whether the second bicuspid is homologous with the third or the fourth premolar. In support of my view, I have in my own practice met with a case in which there were in the lower jaw three bicuspids present *in* the arch on each side, the supernumerary ones being the most distal. That on the one side erupted some time after the extraction of the first molar, that on the other after the removal of the second molar (the first on that side having been long previously removed, the second appropriating most of its site).

A model showing a somewhat similar state of matters is in the Odonto-Chirurgical Society's Museum, but history unknown.

Then I hold that those more or less abnormally formed supernumeraries met with on the buccal surfaces of the molars in the upper jaw, from between the second bicuspid and the first molar, back to the wisdom tooth, and also not unfrequently geminated with the second and third molars, are the missing fourth premolar which has moved backwards with the development backward of the jaw to give room for the permanent molars.

Another point in support of my view which I regard as important, although it is an indirect one, is that one of the normal bicuspids and that *not* the first, is now not unfrequently m t with dwarfed, sometimes very much so, and in one case I have met with it conoid, showing that it, following in the wake of the lateral incisor and the wisdom tooth is marked out for suppression.

I do not suppose that as yet I have much support in my views as to the premolars, still they have received the honor of being noticed in the Manual, although, as yet only in a foot note. I am sorry to say that that volume, able as I regard it, and much as I admire it, shows even in its latest edition many instances of both careless observation and writing, the more to be regretted, seeing that it is not at all likely to have any competitor as a text book in this country at least, and unfortunately the subject does not, so far as I know, form part of the curriculum of any American college (although it finds a place in the "American System of Dentistry").

I will only notice a few of these. "The second deciduous molar has four cusps and resembles a second lower permanent molar." "Cementum; in many teeth of persistent growth it originally invested the whole crown, and after it has been worn from the exposed grinding surface, continues to invest the sides of the tooth, (see the description of the complex teeth of the elephant, cow, horse, &c.)," none of these examples being teeth of persistent growth, and their only claim to complexity lying in their having very long cusps.

We still have the old confusion regarding MM.1 and PM.1, and no notice is taken of the fact that the Tapiridæ are probably the only recent mammals which have both.

In three places, treating of the premolars in the bears, he confounds teeth shed with teeth suppressed, I quote the last. "All four of the premolars seldom persist through the lifetime of the animal, the first premolar, however, is rarely (if ever in recent species) lost, the second being the first to fall out, and then the third. As the fourth is never lost, in most adult bears the first and fourth premolars are found with a wide interval between them. The premolars of bears thus form an exception to the rule, that when a tooth is lost from the premolars the loss takes place from the front of the series." The rule among the carnivora being the incisors and premolars are suppressed from the front, molars from the back of each series.

We have continued the old objections to the term canine being given to a special tooth in the typical dentition. There is no greater need to give the term canine a twofold sense than the term incisor and molar. If canine is merely equivalent to caniniform premolar, what name is to be bestowed on the *temporary* canine?

The central pair of upper incisors in the kangaroo rats, and the lower incisors in the kangaroos are still erroneously given as of persistent growth.

And now, in conclusion, allow me to express my regret that so few of our profession take up comparative dental work, looking upon it as a subject only to be got up for the final and then allowed to evaporate. I think that if they would only interest themselves in the subject a little further, they would find it a source of much pleasure. A little more acquaintance with it would, I think, have made most of the writers on dental pathology hesitate in stating that hypertrophy of the cementum or dental exotosis necessarily involves pain, when they saw that in many mammals the cementum steadily increases in thickness during their whole lifetime quite normally, as well seen in the marine carnivora, and more especially in the smaller teeth of the walrus, in old individuals of which species cementum constitutes by far the larger portion of the tooth. Again the tusks of the same mammal show us that intrinsic calcification of the pulp, that is its conversion into osteodentine, may be going on continuously without involving even the idea of pain.

I now conclude gentlemen, thanking you for your kind attention to what I fear has been a very inadequate address.—*Dental Record.*

